# Safety and efficacy of new integrated bipolar and ultrasonic scissors compared to conventional laparoscopic 5-mm sealing and cutting instruments

**DOI:** 10.1007/s00464-012-2229-0

**Published:** 2012-03-24

**Authors:** Daniel Seehofer, Martina Mogl, Sabine Boas-Knoop, Juliane Unger, Anja Schirmeier, Sascha Chopra, Dennis Eurich

**Affiliations:** 1 Department of General, Visceral and Transplantation Surgery, Charité-Universitätsmedizin Berlin, Campus Virchow-Klinikum, Augustenburger Platz 1, 13353 Berlin, Germany; 2 Department of Laboratory Animal Sciences, Charité-Universitätsmedizin Berlin, Campus Virchow-Klinikum, Berlin, Germany

**Keywords:** Instruments, Technical, Abdominal, Vascular (blood vessels)

## Abstract

**Background:**

Hemostasis is a central issue in laparoscopic surgery. Ultrasonic scissors and bipolar clamps are commonly used, with known advantages with each technique.

**Methods:**

The prototype of new surgical scissors, delivering ultrasonically generated frictional heat energy and bipolar heat energy simultaneously (THUNDERBEAT^®^ [TB]), was compared to ultrasonic scissors (Harmonic ACE^®^ [HA]) and an advanced bipolar device (LigaSure^®^ [LS]) using a pig model. As safety parameters, temperature profiles after single activation and after a defined cut were determined. As efficacy parameters, seal failures and the maximum burst pressure (BP) were measured after in vivo sealing of vessels of various types and diameters (categories 2–4 and 5–7 mm). Moreover, the vertical width of the tissue seal was measured on serial histological slices of selected arteries. The cutting speed was measured during division of isolated arteries and during dissection of a defined length of compound tissue (10 cm of mesentery). Burst-pressure measurement and histological analysis were performed by investigators blinded to the used sealing device.

**Results:**

Using the TB, the burst pressure in larger arteries was significantly higher (734 ± 64 mmHg) than that of the HA (453 ± 50 mmHg). No differences in the rate of seal failures were observed. The cutting speed of the TB was significantly higher than that of all other devices. Safety evaluation revealed temperatures below 100 °C in the bipolar device. The maximum temperature of the HA and the TB was significantly higher. No relevant differences were observed between the HA and the TB.

**Conclusions:**

The ultrasonic and bipolar technique of the TB has the potential to surpass the dissection speed of ultrasonic devices with the sealing efficacy of bipolar clamps. However, heat production that is comparable to conventional ultrasonic scissors should be minded for clinical use.

Effective hemostasis is one of the central issues of laparoscopic surgery. Techniques such as suture ligation, which are easily used in open surgery, are technically demanding and time-consuming if applied laparoscopically. In addition, bleeding might be difficult to control laparoscopically, and a clear view of the bleeding source is often difficult to obtain. Thus, advanced laparoscopic procedures are largely dependent on either mechanical methods of hemostasis (clips or vascular staplers) or on energy-based surgical devices. Nowadays, various different electrosurgical devices are commercially available. For small vessels, monopolar or conventional bipolar electrocautery is often applied and represents basic instruments in laparoscopic surgery. However, for safe dissection of medium-size vessels, advanced bipolar or ultrasonic devices are used among others in colorectal, adrenal, and obesity surgery as well as in urological and gynecological surgery [1–7].

Ultrasonic scissors are known to safely seal arteries of up to 5 mm; likewise, the Harmonic ACE^®^ (Ethicon Endo-Surgery, Cincinnati, OH, USA) is approved for vessels up to 5 mm. Advanced bipolar clamps like ENSEAL^®^ (Ethicon Endo-Surgery) and LigaSure V^®^ (Valleylab Inc., Boulder, CO, USA) are approved for vessels of up to 7 mm in diameter. All of these instruments are known to possess advantages and disadvantages. A major advantage of ultrasonic scissors is the combination of the sealing and cutting step in a single process, leading to faster tissue division and thereby more comfortable preparation in combination with effective sealing. However, maximum temperatures of about 200 °C or even higher at the jaws, e.g., after activation for 10 s, have been described [8]. This entails a certain lateral thermal damage and potential injury to adjacent organs. Therefore, preparation close to susceptible organs requires appropriate attention. Bipolar devices such as ENSEAL^®^ and LigaSure^®^ work with pulsed bipolar energy and a feedback control of the energy output during tissue coagulation. Thus, heat production is lower than that by ultrasonic scissors and the maximum temperature during activation is below 100 °C [9]. Because of the lower temperatures, a major disadvantage of bipolar devices is the lack of simultaneous tissue division. Therefore, most clamps have an integrated cutting blade that mechanically divides the tissue in a second step and thereby markedly prolongs the total dissection time.

The aim of this study was to perform a preclinical in vivo comparison of a new surgical tissue management system, which combines ultrasonic vibration and tissue dissection with bipolar coagulation (THUNDERBEAT^®^, Olympus Medical Systems Corp., Tokyo, Japan), with a conventional ultrasonic scissor and a bipolar vessel clamp, with respect to safety (thermal profile and histological damage) and efficacy (sealing capability and cutting speed).

## Materials and methods

### Animals

Eight German Landrace pigs weighing 45–60 kg were used for the experiments. The pigs were housed at least 1 week before the experiments at the Department of Laboratory Animal Sciences of the Charité and had free access to standard chow and water. The study was conducted in accordance with the German legislation on the protection of animals and was approved by the local authorities (reference number G 0150/10, Landesamt für Gesundheit und Soziales, Berlin, Germany).

### Surgical instruments

**Fig. 1:**
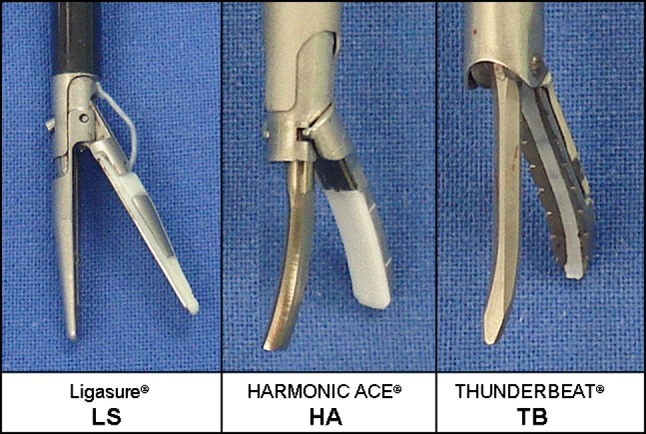
Detailed view of the jaws of the different devices used in the present experiments

**Fig. 2:**
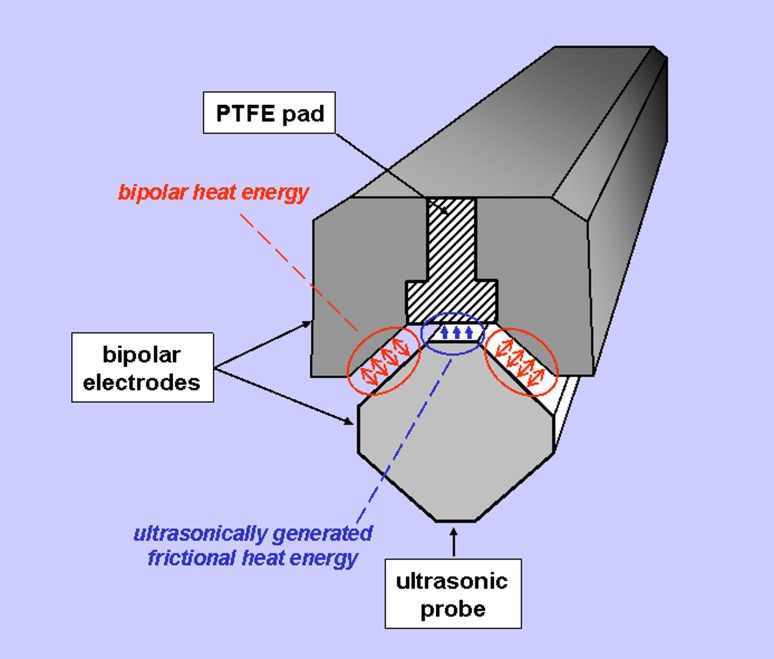
Cross section of the jaws of the TB illustrating its mode of operation. Bipolar energy is delivered laterally (*red arrows*) and ultrasonic energy centrally (*blue arrows*), leading to additional sealing and simultaneous division of the tissue

The following 5-mm laparoscopic sealing and cutting devices were compared with respect to their safety and efficacy: LigaSure^®^ V (Valleylab Inc., Boulder, CO, USA), Harmonic ACE^®^ (Ethicon Endo-Surgery, Cincinnati, OH, USA) and THUNDERBEAT^®^ (Olympus Medical Systems Corp., Tokyo, Japan). For the LigaSure^®^ (abbreviated as LS from hereon) and the Harmonic ACE^®^ (HA), commercially available instruments were used according to the manufacturers’ instructions. For THUNDERBEAT^®^ (TB), a prototype from Olympus Medical was used. The TB device integrates two energy modalities since it delivers both ultrasonically generated frictional heat energy and electrically generated bipolar heat energy simultaneously if used in the “seal-and-cut” mode. The design of the jaws is depicted in Fig. 1. In principle, bipolar heat energy is applied laterally and additional sealing and cutting is achieved by ultrasonic energy centrally (at the region of the white Teflon band, see Figs. 1 and 2). Additionally, a “seal” mode can be activated, leading to delivery of only bipolar energy. However, this mode was not evaluated in the present experiments since tissue division is not possible using this mode. Isolated use of the ultrasonic mode is not possible in this device. For practical reasons, two different devices were used on each animal. The sequence of devices and the application of the sealing instruments on each animal were randomized.

The following a priori hypotheses were investigated in the present experiments (sample size calculations were performed prior to the study based on the results of previous preliminary experiments):

1. The burst pressure of the TB in large arteries (≥5 mm) is superior to that of the HA.
2. The dissection speed of the TB in isolated vessels and compound tissue is superior to that of the LS.
3. The heat production of the TB is clinically comparable to that of the HA.

The primary end point was the mean burst pressure as the parameter of efficacy. Based on the preliminary data of a mean burst pressure of 750 mmHg (TB) and 500 mmHg (HA), a total of 43 seals per device were required to show a statistical significance on a 0.05 level with a power of 0.9. For differences in the speed of dissection of isolated arteries using a single activation, a sample size of 14 was required to detect a difference of 4.0 and 7.5 s. For repeated activation during dissection of compound tissue, a sample size of 8 was required to detect a difference of 20 and 30 s on the same power level. Heat production was investigated in a noninferiority design using a clinically relevant range of 150–250 °C, which is well within the range for different ultrasonic devices reported in the literature [8, 10]. Additional secondary end points such as histological width of the tissue seal were analyzed.

### Cutting speed

The cutting speed was measured during straight dissection of a defined length of the small bowel mesentery (10 cm). Thus, the cut mode of the THUNDERBEAT^®^ was compared with the “max” mode (level 5, Table 1) of the Harmonic ACE^®^. The time until dissection and eventual seal failures were recorded. This procedure was repeated twice per animal and device, resulting in eight measurements per device. In addition, the time for sealing and dissection of the arteries was recorded. The carotid, lienal, femoral, iliac, popliteal, mesenteric, renal, axillar, and brachial arteries were used for determination of the cutting speed followed by burst pressure measurement or histological analyses (see below). For dissection of isolated arteries, the cut mode of the TB was compared with the “min” mode (level 3, Table 1) of the HA and the standard mode of the LS. Time was measured with standard digital stopwatches. Each process was measured simultaneously by two persons and the mean of both measurements was recorded. For all devices, measurement of time was started when the instrument was in place and ended with complete division of the respective tissue. For the bipolar clamp, this means the cumulative time of the sealing and the cutting process.

**Table 1:** Technical specifications of the different devices

	Ultrasonic		Bipolar	
	Frequency	Amplitude	Power (at rated load)	Voltage control
TB Seal and Cut (Level 1)	47 kHz	80 μm	38 W	92 Vp
HA	55.5 kHz	Level 3: 53.4 μm	–	–
		Level 5: 100 μm		
LS (Force TRIAD, Level 2)	–	–	75 W	80 Vp

### Burst pressure measurement

Burst pressure of the sealed vessel segments was measured ex situ. In brief, a catheter was introduced into the open end of the vessel segment and secured. Normal saline was infused into the arterial lumen at a fixed rate (Lambda VIT-FIT, LAMBDA Laboratory Instruments, Zurich, Switzerland), and the pressure was recorded by means of a pressure transducer (Greißinger Electronic GMH3150, Regenstauf, Germany). The maximum pressure (in mmHg) before leakage at the sealing site was defined as burst pressure. In case of leakage from a different site, the vessel was excluded from further analysis. All burst pressure measurements were performed by two persons, blinded for the respective study groups.

### Thermal evaluation

Thermal profiles during and after activation of the scissors were analyzed in detail using two different measuring methods. First, they were measured indirectly using an infrared camera (Variocam T, Jenoptik, Jena, Germany). To avoid reflections and disturbance of the measurement at the metallic parts, all metallic parts on the outer side of the instruments were blackened. Heat production was determined during a single activation of the different devices with dissection of mesentery. All instruments were activated until final tissue division. The devices were fixed at the shaft to avoid movement during measurement. The measured safety parameters were the maximum temperature at the outer side of the jaws and the time to decline to 60 °C after activation.

In addition, for confirmation of the temperatures obtained by indirect measurement (infrared camera), the temperature was directly measured using a thermosensor (K type thermocouple, Qilian Power Equipment, China). However, for technical reasons, the temperature was measured only inside the jaws by grasping the thermosensor with the jaws after cutting the 10 cm of mesentery. This measurement reflects the maximum inside temperature after a longer period of continuous/repeated activation. It might not be directly comparable with the maximum outside temperature, which is supposed to be slightly lower. Again, the maximum temperatures and the time to decline to 60 °C were measured.

### Surgical procedure

First, a midline abdominal incision was performed from the xiphoid process to the symphysis. All experiments started with the measurement of the cutting speed in the mesentery in combination with the thermosensor measurement after cutting 10 cm of mesentery. Afterward, the small bowel was placed in the abdominal cavity and covered with moistened gauze to avoid drying. Isolation of various arteries for sealing and BP measurement was started peripherally. First, separate bilateral incisions were made medially at both hind limbs for dissection of the femoral and popliteal arteries. Next, the front limbs were used for preparation of the axillary and brachial arteries using separate bilateral incisions. This was followed by preparation and sealing of abdominal vessels. Finally, the carotid arteries were prepared bilaterally via a longitudinal median cervical incision. Arterial branches that potentially interfered with the BP measurements were ligated. Before sealing and division of the vessel, the external diameter was determined. The cutting devices were randomized and stratified for the diameter category as described above. Traction on the arteries was avoided during activation of the instruments. To ensure a maximum comparability of the different instruments, only one single seal and cut was used for these experiments. No additional sealing steps next to the cutting site at both ends were performed, although this may be done in clinical practice to increase the width and safety of the seal. In case of primary seal failure, a burst pressure of 0 mmHg was recorded for further analysis of the data. After finishing the arterial seals, thermal camera measurements were performed. The same standardized surgical workflow was used on all animals. All animals were euthanized after completion of all experimental procedures.

### Histological analysis

For histological evaluation, a total of 60 arterial seals in both vessel categories (20 per device) were collected. These specimens were not used for BP measurement and were immediately fixed in formalin. The samples were embedded in paraffin and serially cut in 5-μm sections. Thus, the cutting plane was placed rectangular to the seal for measurement of the sealing width. Staining with hematoxylin and eosin was performed using routine laboratory methods. The extent of adventitial collagen denaturation proximal to the tissue seal and the presence of gas formation caused by tissue boiling were evaluated qualitatively. The perpendicular width of tissue seal was measured in millimeters, beginning from the cut end of the vessel to the point where the vessel walls separated from each other.

For evaluation of the lateral thermal damage and potential damage to adjacent organs, the mesentery of the small bowel was dissected at 0.5 cm from the small-bowel wall. A non-heat-conducting spacer was used for the standardization of the distance. The tissue was fixed, embedded in paraffin, serially cut, and stained with hematoxylin and eosin. Histomorphological analysis investigated the presence of thermal damage to the small bowel. Thus, the relative number of samples with thermal damage and the histological depth of tissue necrosis were evaluated. All histological analyses were performed blinded for the respective study groups.

### Statistical analysis

All values are given as mean and standard error of mean (SEM). For comparisons of continuous variables between groups, a one-way ANOVA was used followed by a Bonferroni post testing. For categorical variables, the χ^2^ test was used. Differences were considered significant if *p* was less than 0.05. All statistical analyses were performed using PASW 18.0 (SPSS, Inc., Chicago, IL, USA).

## Results

In total, 301 arterial vessels were sealed using the three devices. The different vessels used for the burst pressure measurement were equally distributed among the three groups; no significant differences were seen for a single type of vessel. The rate of bleeding after division of isolated arteries in vivo (seal failures) was not significantly different among the devices. The percentage of seal failures correlated with the increase in vessel size.

**Fig. 3:**
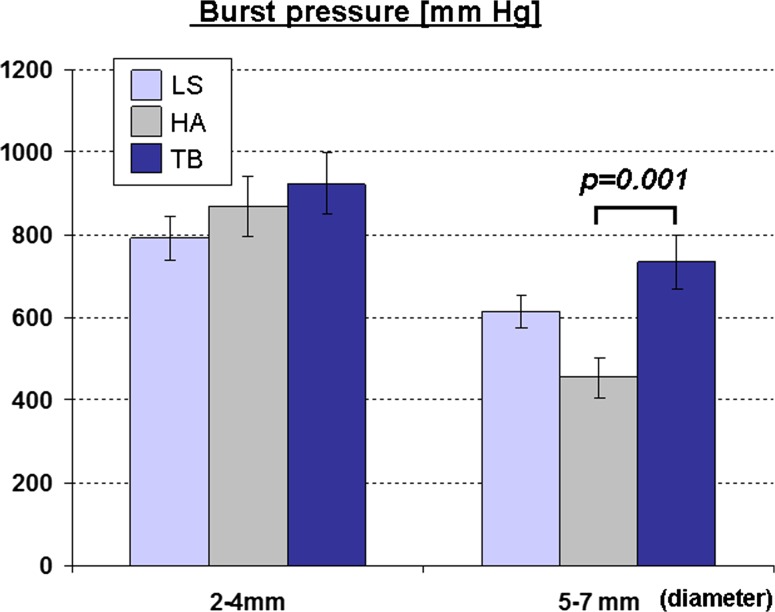
Burst pressure measured after in vivo sealing and division of arteries (*p* values significant by post-hoc comparison are indicated)

*The burst pressure of the TB in the larger-artery category* (5–7 *mm*) *was superior to that of the HA*. The highest mean burst pressure was measured in the TB group (734 ± 64 mmHg); this was slightly higher than in the LS (615 ± 40 mmHg) group and significantly higher than in the HA group (454 ± 50 mmHg, Fig. 3). However, all devices were equally able to reliably seal vessels with a diameter of 2–4 mm with a very high burst pressure and there were no significant differences among the instruments (Fig. 3). Since the additional clinical merit of very high burst pressure values is unclear, the rate of burst pressure values below 300 mmHg, including primary seal failures, was analyzed. This rate was ≤10 % in all devices in small vessels. It increased in the larger-vessel group predominantly for the HA, where the rate of burst pressures below 300 mmHg was 39.5 %, whereas it was significantly lower in the LS (11.1 %) and the TB (10.2 %) group.

**Fig. 4:**
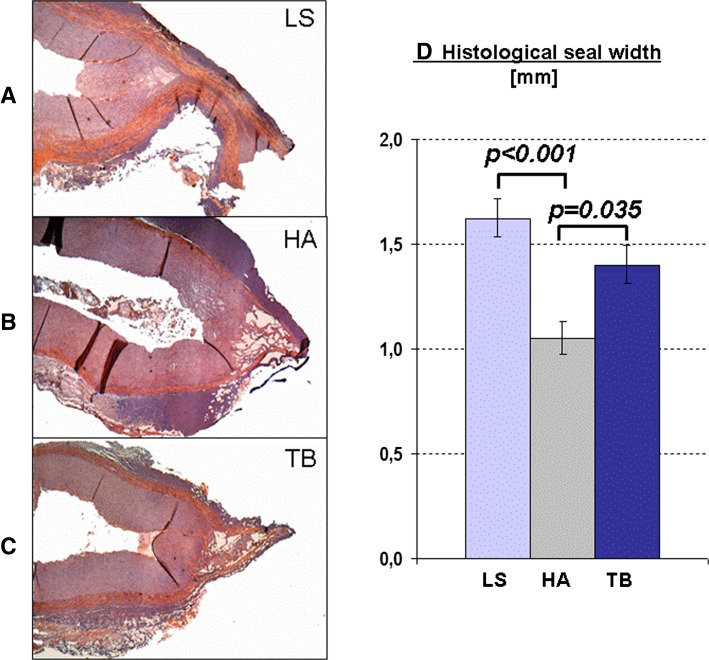
**A–C** Exemplary slides of arterial seals (hematoxylin and eosin stain) showing the seal width and the typical aspect of gas vapor formation, predominantly in the ultrasonic devices (HA and TB). **D** Histological length of the arterial seal (*p* values significant by post-hoc comparison are indicated)

Histological analysis of the seal width as an indirect parameter of seal reliability revealed the broadest seal with the bipolar device (LS). The length of the seal created with the TB was shorter than that of the LS but significantly longer than the seal width of the HA (Fig. 4). Other histological findings were similar in the HA and the TB group. Overall, gas pockets as a particular feature of tissue boiling during dissection were observed mainly in vessels divided with the HA or the TB, and only rarely in vessels sealed with the LS (Fig. 4).

**Fig. 5:**
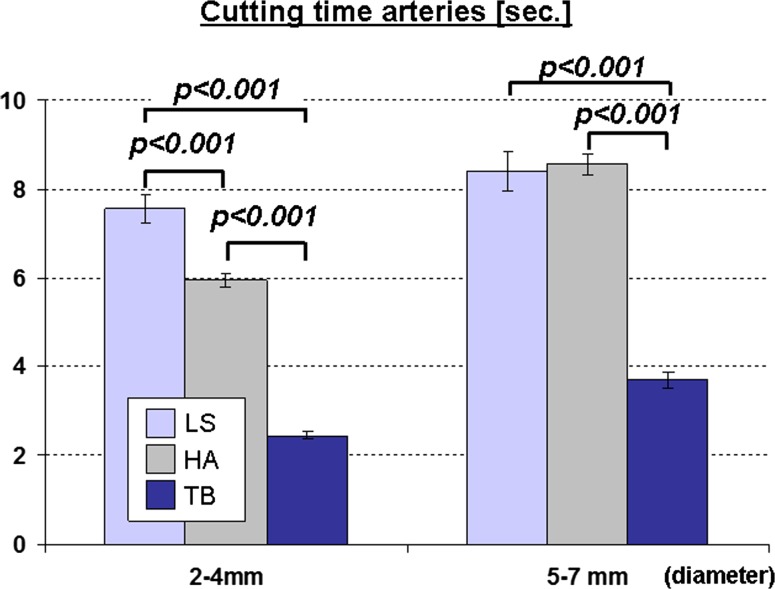
Time needed for division of arteries in both vessel categories (*p* values significant by post-hoc comparison are indicated)

**Fig. 6:**
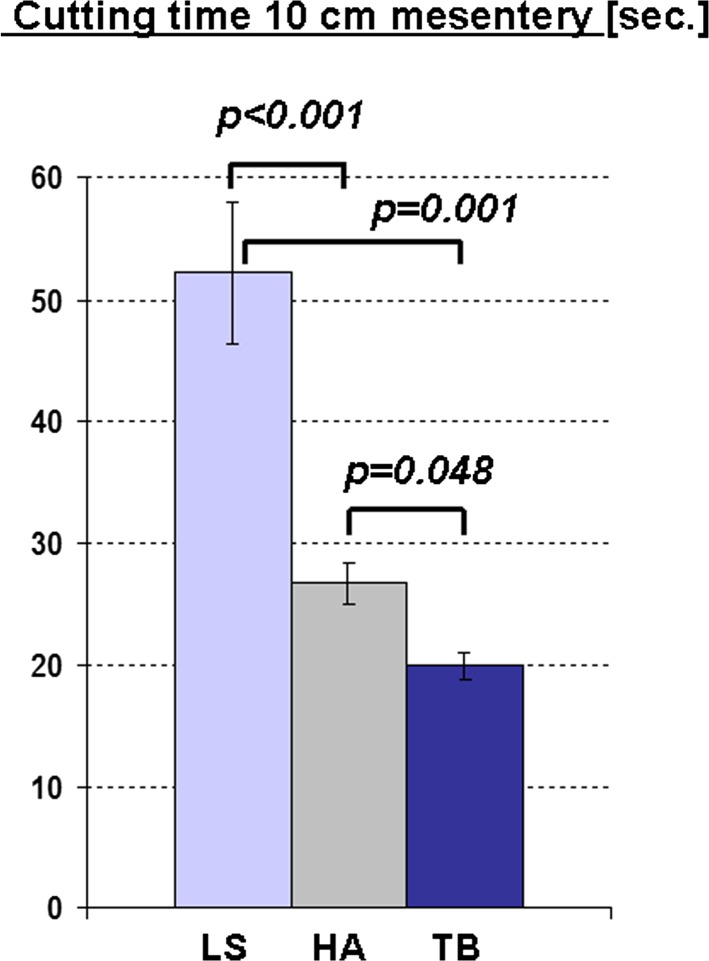
Time needed for sealing and cutting of a standardized length of 10 cm of small bowel mesentery (*p* values significant by post-hoc comparison are indicated)

*The dissection speed of the TB was significantly faster than that of the LS*. The dissection speed for isolated arteries of both size categories (Fig. 5) as well as the dissection speed for compound tissue (Fig. 6) was significantly higher using the TB than for the other devices. Thus, 10 cm of mesentery was divided by the TB in 20 ± 1 s, whereas it took twice as long with the LS (52 ± 6 s). The HA also revealed a markedly slower dissection speed than the TB (Figs. 4, 5). No seal failure was observed with any of the devices during dissection of the small-bowel mesentery.

**Fig. 7:**
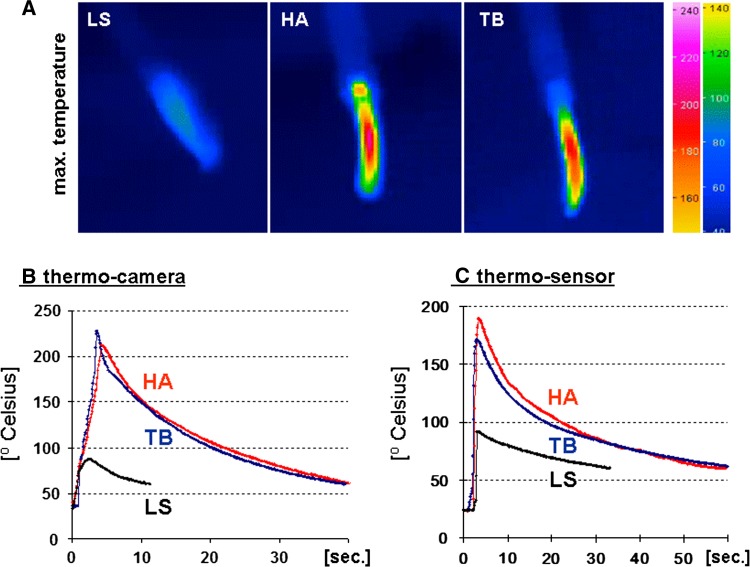
**A** Exemplary thermal camera views of the three instruments at the time of maximum heat production (*upper row*). The color scale encoding the respective temperature (in °C) is depicted on the right-hand side of the figure. **B** Exemplary temperature curves measured with the thermocamera during and after single activation of the devices. **C** Temperature curves of the thermosensor after repeated activation during fast dissection of 10 cm of small bowel mesentery

*Heat production of the TB and the HA was comparable*. The temperature profile of the HA and the TB was similar (Table 2) with respect to the maximum heat production and the kinetics of cooling down to 60 °C (Fig. 7). Moreover, the lateral heat flash was similar between the HA and the TB as shown in exemplary thermal camera shots at the time of maximum temperature (Fig. 7). The maximum temperature during activation and shortly thereafter was around 200 °C in the HA and TB groups. However, the indirect measurements revealed slightly different maximum values and intervals. Whereas the TB had slightly lower values for the thermosensor measurements, the HA had lower values in the thermocamera measurements. However, none of these differences was significant. Apart from small changes in the maximum temperature for view seconds, the further temperature profile was almost identical for the TB and the HA as shown in the exemplary temperature curves in Fig. 7. In contrast, the temperature in the LS group during and after activation was constantly below 100 °C. Lateral thermal damage was investigated in small-bowel specimens after division of the mesentery 5 mm from the bowel wall. No histological damage to the small bowel wall was observed in any device during analysis of serial slides (Table 2). This confirms that 5 mm is a sufficient safety margin for all devices.

**Table 2:** Summary of the safety data*

	LS	HA	TB
(a) Thermosensor			
Maximum temperature^b^ (°C) (95% CI)	86 ± 2^c^ (81–91)	192 ± 7 (175–208)	172 ± 7 (158–187)
Time to decline to 60 °C (s) (95 % CI)	34 ± 3^c^ (29–40)	54 ± 3 (48–60)	60 ± 3 (53–66)
(b) Thermocamera			
Maximum temperature^d^ (°C) (95 % CI)	85 ± 3^c^ (80–90)	209 ± 7 (196–223)	229 ± 9 (209–241)
Time to decline to 60 °C (s) (95 % CI)	8 ± 1^c^ (6–10)	33 ± 1 (31–35)	34 ± 1 (32–36)
(c) Histological damage of small bowel (distance = 5 mm) (*n*)	0/8	0/8	0/8

* (a) Thermosensor: Heat production measured by thermosensor after cutting 10 cm of the small bowel mesentery. (b) Thermocamera: Heat profile during single activation and division of mesenteric tissue determined by an infrared camera. (c) Histological damage of small bowel: Samples with histological damage to the small bowel after standardized division of the small bowel mesentery 5 mm distant to the bowel wall

^b^After repeated activation, see Material and methods

^c^ *p* < 0.05 versus HA and TB

^d^After single activation

## Discussion

Advanced surgical procedures, especially if performed laparoscopically, require electrosurgical instruments that achieve reliable hemostasis and can perform comfortable and fast tissue dissection. Moreover, a favorable safety profile is relevant. Despite continuous progress in the technical development of instruments, all available instruments still have disadvantages. For relevant arteries (≥4 mm), many surgeons still prefer to use additional vascular clips for safety reasons, especially because of a certain rate of seal failures in larger vessels and the resulting bleeding is more difficult to control. Besides surgical clips, advanced bipolar clamps and ultrasonic scissors are most commonly used for hemostasis in laparoscopic surgery.

As shown in the present experiments, the combination of bipolar energy and ultrasonic energy in a single device (THUNDERBEAT^®^) yielded better sealing abilities in comparison with that of a solely ultrasonic device (Harmonic ACE^®^) and increased dissection speed compared to an advanced bipolar clamp (LigaSure^®^). The results of the burst pressure measurements for the HA and the LS were more or less comparable to those obtained in previous experiments [11, 12]. For minor differences, a different setup for burst pressure measurement, different sealing parameters, or other confounding variables might be the cause [13]. Two known confounding factors are the intraluminal hematocrit and the intraluminal protein content, which have been shown to influence the burst pressure after sealing with both the Harmonic ACE^®^ and the LigaSure V^®^ [14]. To definitely exclude nonphysiological conditions or confounding parameters, all sealing procedures in the present study were performed in vivo using a standardized and randomized protocol.

TB has been shown to achieve burst pressures comparable to peak values of mechanical occlusion by surgical clips as reported by Newcomb et al. [15]. In this study, surgical clips achieved a mean burst pressure of 757 mmHg in the large-vessel category of 6–7 mm. Interestingly, most mean burst pressure values obtained by Newcomb et al. were very similar to our results with respect to the devices used in both studies. However, as in most other studies [12, 15], a relatively wide distribution of individual burst pressure data for each device was observed in our experiments as well. One reason might be traction on the arteries during activation of the instruments, especially with ultrasonic devices which are more liable to have this confounding characteristic [12].

The histological length of the seal is often used as a surrogate of its bursting strength, since, according to Laplace’s law, the tension in the vessel walls is dependent on the width of the vascular seal. The seal width of the HA in a previous study was 0.9 mm, which is similar to the 1.0 mm in our experiments [11]. The TB produced a significantly broader seal, which might be based not only on different technology but also on the different designs of the jaws. However, no direct correlation between seal width and burst pressure is possible, but a broader sealing of the tissue is regarded as a prerequisite for a reliable seal. Nevertheless, the surgically relevant functional parameter is the burst pressure (see above). The seal width is influenced mainly by the width of the jaws of the respective device. Since the bipolar clamp (LS) has broader jaws than the HA and the TB, it revealed the longest seal width. However, during meticulous surgical preparation, broader jaws might not be advantageous.

In our preclinical study, the markedly increased dissection speed and efficacy of the TB was not associated with changes in safety parameters compared to the HA. Similar results for all safety parameters were obtained in the HA group and the TB group using two different measuring methods (infrared camera and thermosensor). Thus, the general temperature kinetics of the conventional instruments in our series confirmed previously reported data. In accordance with our findings, Kim et al. [10] reported temperatures around 200 °C during and shortly after activation. Importantly, the time to decline to 60 °C in our experiments correlated with the time of activation. Again, direct comparison of the TB and the HA revealed no clinically relevant differences after short and continuous activations. In accordance with our data, the HA requires almost twice the time to cool down to 60 °C than bipolar devices. The same is true for the TB. Therefore, from the safety point of view, the “seal-and-cut” mode of the TB, which was used in the present experiments, is to be handled clinically like ultrasonic scissors. Since the Harmonic ACE^®^, which produces a comparable heat profile, has been used regularly in clinical procedures for many years, this level of heat production is acceptable. For ultrasonic devices a temporary heat production of more than 200 °C and a certain lateral damage of 2–3 mm is a well known and clinically accepted phenomenon [8, 16]. Even a maximum temperature of 294 °C has been reported in an experimental study using Ultracision^®^ [8]. These differences in heat production between ultrasonic and bipolar clamps are a consequence of different purposes. Tissue sealing is achieved at temperatures of around 100 °C, whereas (nonmechanical) cutting requires temperatures of around 150–200 °C [17, 18]. Therefore, the increased dissection speed is inevitably associated with higher temperatures. A fast tissue dissection is important since operating time is nowadays an important economic factor, in open [19, 20] and laparoscopic surgery [1, 21].

Another point that needs to be addressed is that the sealing procedures were not performed laparoscopically, though the devices used are designed for laparoscopic surgery. However, this basic preclinical evaluation was to be a standardized comparison of these instruments with respect to efficacy and safety parameters, including an evaluation of heat production. For technical reasons and standardized evaluation, this is possible only with open surgery.

In conclusion, all tested devices were equally able to safely divide arteries of up to 4 mm in diameter. From the studied devices, the THUNDERBEAT^®^ had the fastest dissection in combination with the highest burst pressure values, even in vessels measuring 5–7 mm in diameter. For technical reasons, heat production in ultrasonic cutting devices was higher than in bipolar devices. Therefore, from the point of view of safety, the TB should be handled like ultrasonic scissors. According to the present data, it has the potential to surpass the dissection speed of ultrasonic devices with the sealing efficacy of bipolar clamps.
